# Characterization and structural determination of a new anti-MET function-blocking antibody with binding epitope distinct from the ligand binding domain

**DOI:** 10.1038/s41598-017-09460-2

**Published:** 2017-08-21

**Authors:** Danielle M. DiCara, Dimitri Y. Chirgadze, Anthony R. Pope, Aneesh Karatt-Vellatt, Anja Winter, Peter Slavny, Joop van den Heuvel, Kothai Parthiban, Jane Holland, Len C. Packman, Georgia Mavria, Jens Hoffmann, Walter Birchmeier, Ermanno Gherardi, John McCafferty

**Affiliations:** 1 MRC Centre, Hills Road, Cambridge, CB2 2QH UK; 2 Department of Oncology, University of Cambridge, Cambridge Biomedical Campus, Cambridge, CB2 0XZ UK; 3 Department of Biochemistry, University of Cambridge, 80 Tennis Court Road, Cambridge, CB2 1GA UK; 4 IONTAS Ltd, Babraham Institute, Babraham, Cambridgeshire CB22 3AT UK; 5 Helmholtz Zentrum für Infektionsforschung, Inhoffenstraße 7, 38124 Braunschweig, Germany; 6 Max Delbrueck Center for Molecular Medicine (MDC) in the Helmholtz Association, 13125 Berlin, Germany; 7 Leeds Institute of Cancer and Pathology, University of Leeds, St James’ University Hospital, Beckett Street, Leeds, LS9 7TF UK; 8 Experimental Pharmacology & Oncology Berlin-Buch GmbH, Robert-Rössle-Str. 10, 13125 Berlin-Buch, Germany; 9 Division of Immunology and General Pathology, Department of Molecular Medicine, 1 via A Ferrata, 27100 Pavia, Italy; 10 Present Address: Genentech Inc., South San Francisco, 94080 USA; 11 Present Address: Faculty of Natural Sciences, Keele University, Staffordshire, ST5 5BG UK

## Abstract

The growth and motility factor Hepatocyte Growth Factor/Scatter Factor (HGF/SF) and its receptor, the product of the *MET* proto-oncogene, promote invasion and metastasis of tumor cells and have been considered potential targets for cancer therapy. We generated a new Met-blocking antibody which binds outside the ligand-binding site, and determined the crystal structure of the Fab in complex with its target, which identifies the binding site as the Met Ig1 domain. The antibody, 107_A07, inhibited HGF/SF-induced cell migration and proliferation *in vitro* and inhibited growth of tumor xenografts *in vivo*. In biochemical assays, 107_A07 competes with both HGF/SF and its truncated splice variant NK1 for MET binding, despite the location of the antibody epitope on a domain (Ig1) not reported to bind NK1 or HGF/SF. Overlay of the Fab-MET crystal structure with the InternalinB-MET crystal structure shows that the 107_A07 Fab comes into close proximity with the HGF/SF-binding SEMA domain when MET is in the “compact”, InternalinB-bound conformation, but not when MET is in the “open” conformation. These findings provide further support for the importance of the “compact” conformation of the MET extracellular domain, and the relevance of this conformation to HGF/SF binding and signaling.

## Introduction

A major challenge in the therapy of solid tumors, and notably of carcinomas that constitute over 85% of all human cancers, is the development of agents that inhibit metastasis, namely the growth of cells derived from the primary tumor at distant sites in the body. Metastasis is a multi-stage process in which cancer cells migrate into adjacent tissues (invasion), cross the wall of blood or lymphatic capillaries to be transported across the general circulation (intravasation), exit from the bloodstream into a secondary tissue/organ (extravasation) and finally give rise to secondary tumors^1^. In principle, each of these stages may be targeted in therapy, and the ability to target multiple stages simultaneously is an attractive prospect.

The signaling system mediated by the growth and motility factor HGF/SF and its receptor MET, the tyrosine kinase encoded by the *MET* proto-oncogene, has multiple and essential physiological roles in vertebrate embryogenesis, where it is required for normal development of the liver parenchyma and the labyrinth layer of the placenta^2, 3^ as well as distant migration of the myogenic precursor cells^4^. HGF/SF and MET have further and important physiological roles in postnatal life, where they control the regeneration after injury of several epithelial organs including skin^5^ and liver^6, 7^. They also exert, however, multiple and crucial roles in the early stages of metastasis of epithelial cancers by controlling (**i**) delamination of epithelial cells and the process of epithelial-mesenchymal transition crucial for long distance epithelial cell migration^4, 8^, (**ii**) degradation of the basement membrane and remodeling of the extracellular matrix *via* urokinase^9^ and matrix metalloproteinases^10^, (**iii**) integrin-dependent migration of cancer cells as a result of activation of focal adhesion kinase and paxillin^11^, (**iv**) formation of the pre-metastatic niche *via* tumor-derived exosomes^12^, (**v**) tumor lymphangiogenesis^13, 14^, a process essential for lymphatic metastasis and, (**vi**) haemangiogenesis^15, 16^. Further, there is growing evidence for a major role of HGF/SF and MET in the maintenance of cancer stem cells in colon^17^, breast^18^ and prostate^19^ carcinomas, and accumulating reports of involvement of the MET-HGF/SF axis in cancer cell resistance to targeted therapies both *in vitro* ^20–23^ and in cancer patients^24–26^.

The MET receptor is a single-pass transmembrane protein consisting of a large extracellular ectodomain, a transmembrane segment and an intracellular receptor tyrosine kinase domain^27^. The ectodomain is a heterodimer arising from furin cleavage of a single precursor chain and consists of an N-terminal 283 amino acid α-chain and the 625 extracellular amino acids of the β-chain. An N-terminal semaphorin homology domain (SEMA), which binds the ligand HGF/SF, is followed by a “stalk” comprising one cysteine rich (CR) domain and 4 immunoglobulin domains termed Ig1-Ig4^28^. The MET ectodomain has been expressed recombinantly as a series of truncations containing the SEMA, CR and 0, 2 or 4 Ig domains, which we will refer to as MET567, MET741 and MET928, respectively. HGF/SF consists of an amino terminal domain, 4 kringle domains (K1 - K4) and a C terminal serine protease homology domain (SPH domain). The N terminal and first kringle domain (NK1) of the HGF/SF ligand drive a high affinity interaction with MET^29^, and the SPH domain also binds MET^30^. The SEMA domain has been reported as necessary and sufficient for HGF/SF binding^28^, although an interaction with the Ig3-Ig4 “lower stalk” region has also been reported^31^.

Here, we report the isolation of a new human, phage-derived monoclonal antibody to MET (107_A07) displaying potent receptor antagonistic activity, and we describe the activity of 107_A07 on cancer cells *in vitro* and in *in vivo*. We also report a crystal structure of the 107_A07 Fab in complex with a MET receptor fragment containing amino acids 519–740, which includes the CR, Ig1 and Ig2 domains but not the ligand-binding SEMA domain. These data provide insights into the conformation of MET during ligand binding and cell signaling.

## Materials and Methods

### Protein production and purification

Soluble MET741 protein^28^ was produced from CHO Lec3.2.8.1 cells and purified by affinity chromatography (NiNTA Superflow, Qiagen) followed by cation exchange (MonoS, GE Life Sciences). 107_A07 and D1.3 Fabs and IgG –formatted antibodies were produced by transfection of suspension HEK293F cells (Invitrogen) with Valproic Acid (Sigma) added to 4 mM following transfection. Fab proteins were purified using affinity resins KappaSelect and/or GammaBind Plus (GE Life Sciences). IgG formatted antibodies were purified by Protein A affinity chromatography. For cell cycle analysis, 107_A07 and D1.3 Fabs were further purified by gel filtration chromatography (Superdex 200 10/300 (GE Life Sciences). Unless stated otherwise, 7A2 scFv was produced in *Pichia pastoris* and purified by Ni-NTA chromatography followed by gel filtration. Anti-MET antibody 5D5 sequences were obtained from US Patent No. 7,476,724 B2. Heavy and light chains were synthesized (GeneArt, Thermo Fisher Scientific) with restriction sites that allowed cloning into Fab vectors pBIOCAM1-3F and pBIOCAM3-3F, expressed in HEK293F cells and the Fab purified by Ni-NTA chromatography.

### FAb PEGylation

Recombinant Fab were partially reduced with TCEP and PEGylated with maleimide-activated PEG (Sunbright ME-200 MAOB or Sunbright ME-200MA3, NOF Europe). PEG-Fab and free PEG were monitored by SDS-PAGE and barium chloride & iodine staining^32^.

### Isolation & affinity maturation of functional MET-blocking antibodies by phage display

Biopanning with a scFv phage library^33^ was performed on solid-phase recombinant MET928 and light chain shuffling performed on the output by cloning the resulting VH gene pool back into the original scFv phage library^34^. Biopanning with the chain-shuffled library (10^9^ clones) was performed with biotinylated MET928 in solution and streptavidin-coated dynabeads. Phage pools were cloned into expression vector^35^ and small-scale expressions performed in BL21 (DE3) bacteria in 96-well format. Approximately 960 supernatants were screened directly for inhibition of HGF/SF-induced scatter of BxPC-3 human pancreatic cancer cells. Affinity maturation was performed by diversification of the CDR3 regions of 7A2 VH and VL using oligonucleotide-directed mutagenesis and stringent selection of the resulting phage library with biotinylated MET928 in solution.

### *In vitro* cell-based assays

HGF/SF-induced cell migration across a porous membrane coated with 100 µg/ml collagen (Purecol, Nutacon) was analyzed using a modified Boyden chamber assay. Cells (SKOV-3 or U87MG) were labeled with the fluorescent dye Calcein AM (Life Technologies) and migration assessed by quantification of fluorescence on the underside of the membrane using a Typhoon instrument (GE Life Sciences). Data were analysed with ImageQuant software and background fluorescence subtracted. For cell cycle analysis, U87MG human glioblastoma cells were serum-starved for 48 hours prior to a 24 hour incubation with 300 pM HGF/SF with or without 0.9 µM 107_A07 FAb or 1 µM D1.3 FAb. Cells were trypsinised, fixed, stained with propidium iodide in the presence of RNAse and analyzed by flow cytometry according to standard procedures. *In vitro* angiogenesis assay was performed using the modified co-culture assay as described previously^36^. Briefly, fibroblast cells were seeded in gelatin-coated chamber slides. Human umbilical vein endothelial cells (HUVECs) were seeded on to the confluent fibroblasts and D1.3 and 107_A07 antibodies (200 nM) were added to the cells. The co-cultures were fixed and stained for CD31. Number of tubules was counted manually from 10 fields for each well and the field area was measured using AngioSys 1.0 imaging software.

### Tumor xenografts

NMRI *nu/nu* mice (Crl:NMRI-*Foxn1* ^*nu*^) were obtained from Charles River Laboratories (Sulzfeld, Germany). U87MG cells were obtained from ECACC and identity checked at the DSMZ. Mice were injected subcutaneously with 10^7^ U87MG human glioblastoma cells and antibody administered every 3–5 days between day 7 and 33, when treatment stopped. Groups of 8 mice were treated with 107_A07 IgG at either 2 mg/kg or 10 mg/kg. Control animals were treated with either PBS or D1.3 IgG at 10 mg/kg. All animal experiments were carried out in accordance with the United Kingdom coordinating committee on cancer research regulations for the welfare of animals and the German Animal Protection Law, and were also approved by the local responsible authorities Landesamt für Gesundheit und Soziales Berlin (LAGeSo).

### Surface Plasmon Resonance (SPR) studies

K_D_ was determined using a Biacore instrument and represents the mean of three experiments using 107_A07 FAb purified sequentially on both KappaSelect and GammaBind Plus chromatographic resins. For competition analysis by SPR, a CM5 chip coated with MET928 was exposed to: 7A2 scFv (134 nM, 268 nM), NK1 (238 nM, 476 nM) or a mixture of the two (134 nM 7A2 scFv, 238 nM NK1).

### Competition analysis by ELISA

Fabs were mixed with 100 nM MET928 one hour before addition to microplates coated with HGF/SF or the fragment NK1. After one hour bound MET928 was detected with anti-5xHis (Qiagen) followed by DELFIA® Eu-N1 rabbit anti-mouse-IgG. DELFIA® enhancement solution was then added and signal quantitated using time-resolved fluorescence with a Fusion instrument (Perkin Elmer).

### Complex formation analysis

Fab and MET proteins were co-incubated at a 3:2 molar ratio for 140 minutes at room temperature in 25 mM Tris pH 7.4, 150 mM NaCl, centrifuged and analyzed by size exclusion chromatography (Superdex 200 10/300).

### Co-crystallization of MET/Fab complex

Briefly, Fab 107_A07 and his-tagged MET741 were co-incubated, then digested with Pepsin and EndoHf deglycosidase. The remaining complex was purified by Ni-NTA affinity resin and size exclusion chromatography, concentrated to 5.9 mg/ml and crystallized by the sitting-drop vapor diffusion method at 19 °C.

### Data collection & model generation

X-ray data collection experiments were performed at the European Synchrotron Radiation Facility (Grenoble France), beamline ID29. The crystals diffracted to a maximum resolution of 2.6 Å. The crystals contained two molecules of the MET/Fab complex in the asymmetric unit. The crystal structure was solved using the Molecular Replacement (MR) method. After several rounds of manual rebuilding and refinement the R/R_free_ values reached 21.5% and 25.7% respectively. The coordinates of the structure have been deposited to the Protein Data Bank under accession ID 5LSP. Additional method information is provided in the Supplementary Information file.

### Additional Disclosures

D.D.C., A.P., J.M.C. and E.G. are co-inventors on a patent application relating to the antibodies described in this manuscript. D.D.C. is currently an employee of Genentech, Inc.

## Results

### Generation and affinity maturation of a high affinity antibody blocking MET signaling

Recombinant antibodies recognizing MET928 were generated by phage display using two rounds of panning on immobilized antigen. Binders were generated using a previously described human antibody phage display library of ~10^10^ clones formatted as single chain Fvs (scFvs)^33^. The selected population was affinity matured *en masse* by chain-shuffling the selected population of VH genes^34^. A chain-shuffled scFv library of 10^9^ clones was constructed and was subjected to stringent selection using biotinylated MET928 to allow the emergence of new VH/VL combinations with potentially superior binding properties to the original. In addition to affinity-based approaches, a “competitive elution” selection was performed in parallel in which MET-bound phage populations were incubated with high (micromolar) concentrations of the ligand HGF/SF in an effort to elute HGF/SF-competitive antibodies. Selected populations were cloned into a bacterial expression vector^35^ and individual clones were screened directly for functional activity using a colony scatter assay. Hits emerging from this screen were assessed in a more quantitative cell migration assay, based on migration of fluorescently labeled SKOV3 human ovarian cancer cells using a Boyden chamber. A number of clones were identified that inhibited HGF/SF-induced migration of SKOV3 cells, including clone 7A2 (Supplementary Figure S1) which emerged from “competitive elution” selections. This clone was antagonistic for ligand-induced MET activation but also exhibited a degree of agonism when tested in the absence of added ligand. Specifically, a “bell-shaped” curve, similar to that seen with the natural ligand, was observed with peak agonistic activity around 1 nM 7A2 scFv and a maximum peak height which was lower than that induced by HGF/SF (Fig. S1). It has previously been shown that anti-Met antibodies presented in a bivalent format can display agonistic activity^37^. It is also known that scFv molecules can form dimers and other higher order structures. Separation of monomeric scFv from larger species revealed that agonistic activity in scFv preparations was associated with larger, non-monomeric species (data not shown). Fab are much less likely than scFv to form higher-order species, and so we converted 7A2 to a Fab format.

Conversion of 7A2 to a Fab format diminished the agonistic activity as expected but also resulted in reduced affinity and potency, therefore an additional round of affinity maturation was conducted using oligonucleotide directed mutagenesis to create phage display libraries where the CDR3 region of the V gene heavy chain (VH) was diversified. Mutagenic primers with contiguous blocks of 5–6 randomized amino acids using 3 overlapping primers were used to randomize the 11 amino acids of the VH CDR3 and 9 amino acids from VL CDR3. Stringent selections were carried out using limiting amounts of biotinylated MET928 down to 10 pM. DNA from the output of the resulting selections was subcloned into a bacterial expression vector and 1152 clones were screened by ELISA and sequenced. 146 clones with unique sequences that were positive in ELISA were identified for further study. VL mutants were not identified, suggesting this library was ineffective or was dominated by the parental sequence. The selected clones were expressed, purified and the off-rates compared to the parental 7A2 clone using surface plasmon resonance (SPR). The top 8 clones showing the greatest improvement in off-rate were identified and produced as Fab fragments. Sequence analysis revealed that the top group of clones came from VH CDR3 mutagenesis and all carried a Y105W mutation. In addition, 6/8 of these clones retained G106 and M107 despite the complete diversification within this region of the selected clones. Based on affinity and potency rankings, the affinity-matured clone 107_A07 was selected for further biological and biochemical analysis. SPR experiments yielded a K_D_ in the nanomolar range (3.2 ± 0.7 nM; mean ± SD, three experiments).

### Antibody 107_A07 inhibits migration and proliferation of cancer cells and endothelial tubulogenesis

**Figure 1:**
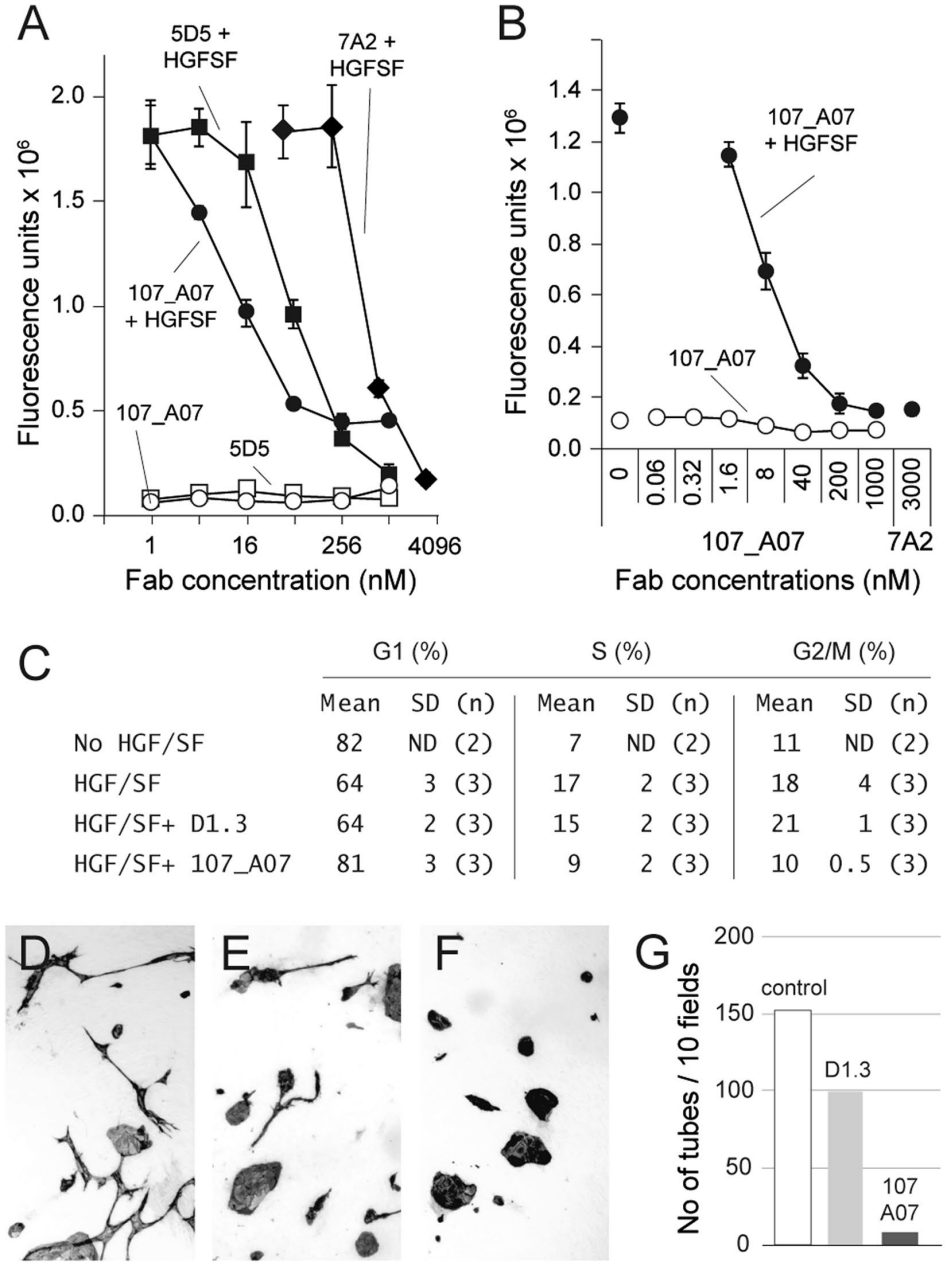
107_A07 FAb inhibits HGF/SF-induced cancer cell migration, DNA synthesis and angiogenesis *in vitro*. Effect of parental Fab-formatted 7A2 and matured 107_A07 Fab on SKOV3 (**A**) and U87MG (**B**) cell migration in the presence and absense of HGF/SF in a modified Boyden chamber assay. A Fab-formatted version of the anti-MET antibody 5D5 was used as a control. Closed and open symbols show respectively the effect of antibody in the presence and absence of HGF/SF. Data represent mean ± standard deviation of triplicate wells. (**C**) Cell cycle analysis by propidium iodide staining of fixed, serum-starved U87MG cells 24 hours after exposure to 300 pM HGF/SF and/or Fab as indicated. Mean and standard deviation of (n) experiments are shown. (**D**,**E**,**F** and **G**) PEGylated 107A07 blocks angiogenesis in an *in vitro* angiogenesis assay in which co-culture with fibroblasts promotes endothelial cell sprouting and tubulogenesis. (**D**) control culture, no Fab, (**E**) PEG-D1.3 Fab, (**F**) PEG-107_A07 Fab, (**G**) quantification of endothelial tubules three days after the addition of Fab.

HGF/SF-mediated activation of MET causes cell proliferation, cell migration, and angiogenesis, all of which contribute to cancer progression^38^. We assessed 107_A07 Fab for inhibition of these processes. Inhibition of cell migration was compared with 7A2, the parental antibody of 107_A07, and as a control we also included a Fab-formatted version of 5D5.v1, the monoclonal antibody from which onartuzumab was derived^37^. 107_A07 Fab dose-dependently inhibited HGF/SF-induced cell migration of SKOV-3 human ovarian cancer cells (Fig. 1A) and U87MG human glioblastoma cells (Fig. 1B).

A degree of agonism observed at higher concentrations with Fab formatted 107_A07 was lost following purification of monomers by size exclusion chromatography, suggesting that the presence of low concentrations of multimeric material could drive agonism (Figure S2). To assess antibody activity on HGF/SF-induced cell proliferation, Fab were further purified by size exclusion chromatography and serum-starved U87MG cells were incubated for 24 hours with HGF/SF in the presence of 107_A07 Fab or a control Fab (humanized anti-hen egg lysozyme, D1.3^39^), followed by DNA content analysis by propidium iodide staining. U87MG cells express HGF/SF^40^ and divide rapidly in culture, however addition of exogenous HGF/SF caused a further and measurable increase in DNA synthesis (Fig. 1C). Treatment with 107_A07 Fab but not control (D1.3) Fab reduced the percentage of cells in S-phase and G2/M phase to basal levels, with a corresponding increase in cells in G1 (Fig. 1C).

We also investigated PEGylation of the 107_A07 Fab, since PEGylation can be used to extend Fab pharmacokinetics *in vivo*. PEGylation of 107_A07 Fab was achieved by covalent attachment of 20 kD polyethylene glycol (PEG) chains to the C-terminal cysteine residues^41^. The majority of unconjugated PEG was removed by size exclusion chromatography and ultrafiltration. In SDS-PAGE, PEG-107_A07 Fab chains displayed reduced migration and insensitivity to reducing agents, consistent with removal of the inter-chain disulphide bond (Fig. S3). PEG-107_A07 Fab retained the ability to inhibit HGF/SF-induced cell migration and demonstrated essentially complete inhibition of cell migration at high concentrations (Fig. S4). In a fibroblast/endothelial cell *in vitro* model of angiogenesis in which fibroblast-derived HGF/SF induces human umbilical vein endothelial cell (HUVEC) tubulogenesis (a process that recapitulates *in vitro* the sprouting of new capillaries *in vivo*) (Fig. 1D), PEG-107_A07 Fab inhibited tubule formation (Fig. 1F and G).

### 107_A07 IgG inhibits the growth of human tumor xenografts *in vivo*

**Figure 2:**
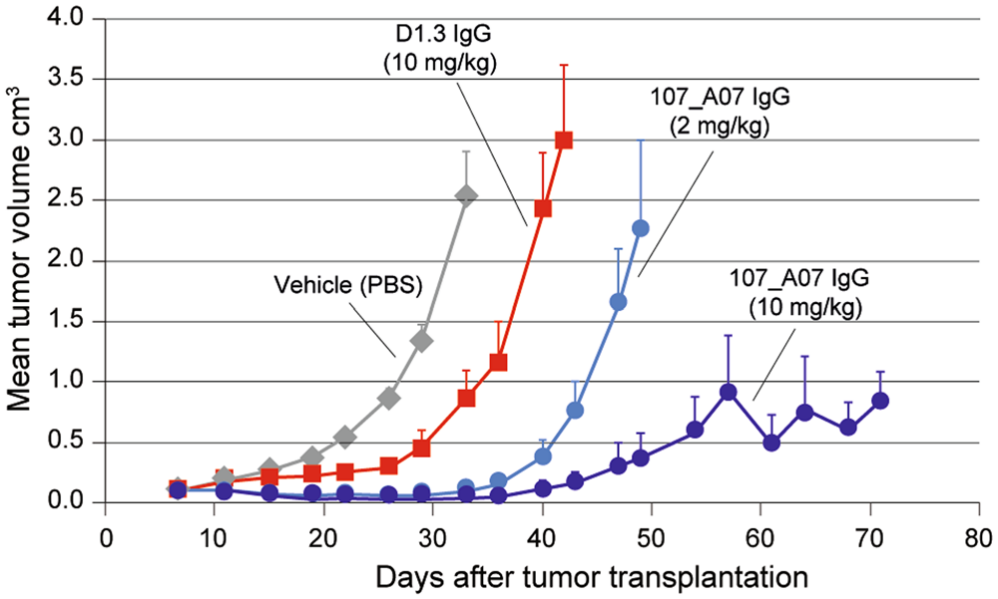
Effect of 107_A07 IgG on the growth of subcutaneous U87MG xenografts. Following xenograft implantation on day 0, mice (n = 8) were given vehicle alone (PBS), control D1.3 IgG (10 mg/kg) or 107_A07 IgG (2 or 10 mg/kg) intraperitoneally at day 7 and then every 3–5 days for 26 days. Tumor volumes were measured by caliper twice weekly.

The VH and VL domains of 107_A07 were expressed as an intact human IgG and the activity of 107_A07 IgG and control (D1.3) IgG were assessed in cell migration assays where agonism was found with 107_A07 IgG (Fig. S5). The peak of agonistic activity was again found to occur at low nanomolar concentration (as with the scFv, Fig. S1) with net antagonism found at higher concentrations (Fig. S5). 107_A07 IgG was administered over 26 days in a human U87MG glioblastoma cell xenograft model and inhibited tumor growth very effectively for up to 70 days at 10 mg/kg 107_A07 IgG (Fig. 2). Thus intact 107_A07 IgG inhibited tumor growth despite its bivalent format (Fig. 2), presumably because an IgG concentration was maintained where net antagonism was achieved.

### 7A2/107_A07 binds within the Ig1-Ig2 domains of MET and competes with HGF/SF in biochemical assays

**Figure 3:**
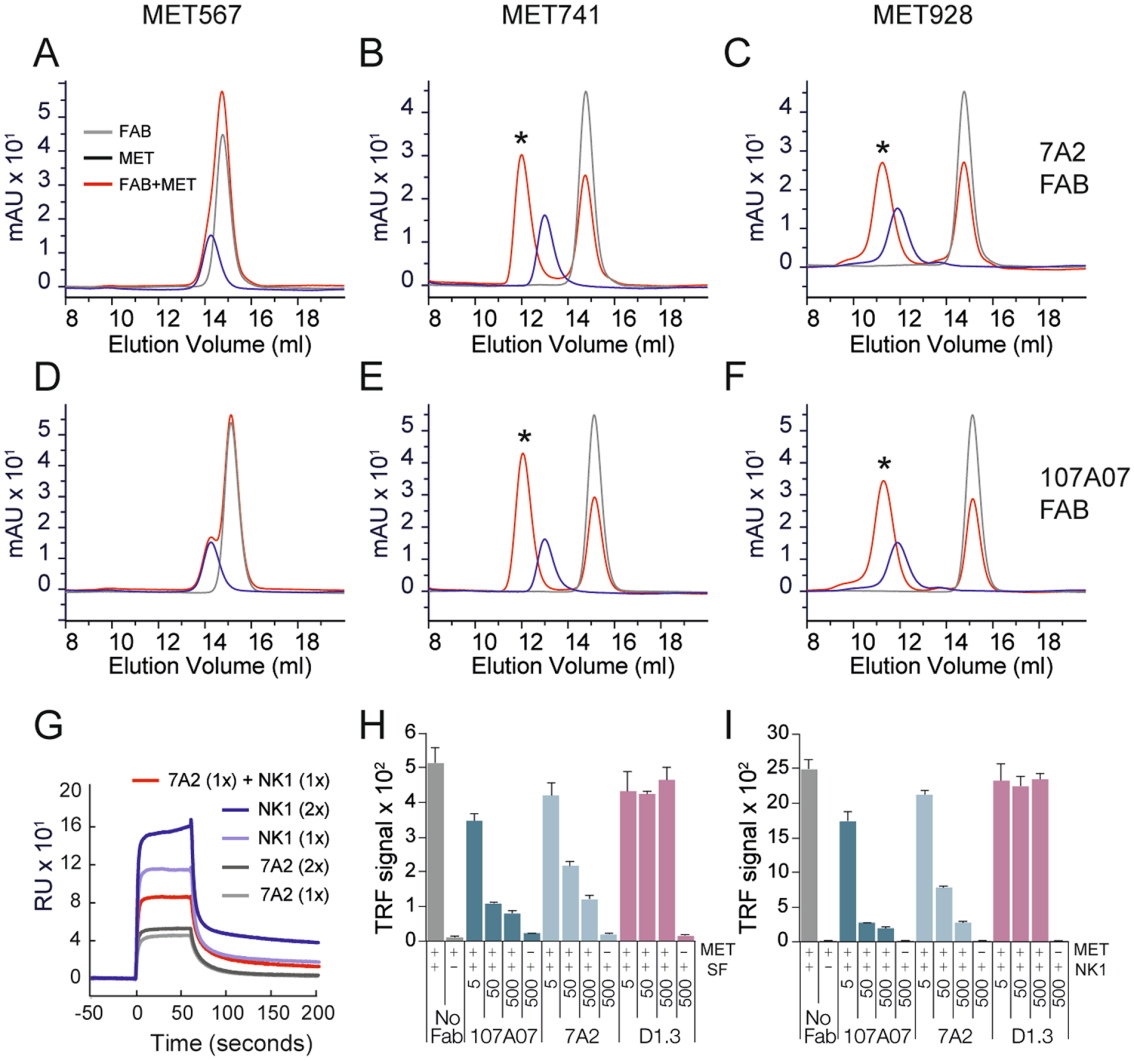
7A2 and 107_A07 bind within the Ig1-Ig2 domains of MET and compete with NK1 for MET binding. Panels A–F show size exclusion chromatography analysis of 6 µM Fab 7A2 (**A**–**C**) or 107_A07 (**D**–**F**) and 4 µM MET567 (**A**,**D**), MET741 (**B**,**E**) or MET928 (**C**,**F**), alone or following co-incubation. Y-axis indicates absorbance at 280 nm and x-axis elution volume. Dark blue line, MET alone; gray line, Fab alone; red line, MET/Fab mixture. Peaks present only in MET-Fab mixtures represent complex formation and are marked with an asterix. (**G**) Binding to a MET928-coated CM5 Biacore chip of 7A2 scFv (light gray line, 134 nM), NK1 (light blue line, 238 nM), or 7A2 scFv mixed with NK1 (red line). Binding of higher concentrations of 7A2 scFv (dark gray line, 268 nM) and NK1 (dark blue line, 268 nM) are also shown for comparison. Sensorgrams are shown aligned to the start of each 60 s injection. (**H** and **I**) Following co-incubation, untagged Fab and His-tagged MET928 were exposed to plates coated with HGF/SF (**H**) or NK1 (**I**) and bound MET928 detected with anti-5xHis peroxidase (Qiagen). Data represent mean and standard deviation of a minimum of three replicates per sample. Fab were tested at 5, 50 and 500 nM.

Initial insights into the 7A2/107_A07 epitope and inhibitory mechanism were obtained by physicochemical studies. 7A2 Fab was incubated with three soluble fragments of the MET ectodomain: MET567, a fragment comprising the SEMA and cysteine-rich (CR) domain, MET741 containing the SEMA, CR and Ig1-2 domains and MET928 containing the SEMA, CR and Ig1-4 domains^28^. Complex formation was assessed by size exclusion chromatography and was readily detectable with constructs MET741 and MET928 but not with MET567 (Fig. 3A–C), indicating that the 7A2/107_A07 epitope is contained within the first two Ig-like domains of the MET stalk structure^28, 42^. Similar results were observed with 107_A07 Fab (not shown). In contrast, a Fab-formatted version of the anti-Met antibody 5D5 was found to bind all 3 fragments (not shown), consistent with binding to a distinct site within the ligand binding SEMA domain^37^. We next investigated whether 7A2/107_A07 competes with HGF/SF or fragments of HGF/SF for binding to MET using SPR. Due to the rapid binding kinetics observed for 7A2, we compared binding of 7A2 and NK1 to immobilized MET extracellular domain (MET928) individually and when mixed. Clear competition for binding was observed with the mixture of 7A2 and NK1 (Fig. 3D). Biochemical competition between 7A2 and NK1 was confirmed by solid phase assays in which pre-incubation of MET928 with either 7A2 or 107_A07 inhibited MET binding to both HGF/SF and NK1 (Fig. 3E and F).

### Crystal structure of the 107_A07 and MET-107_A07 complex

**Figure 4:**
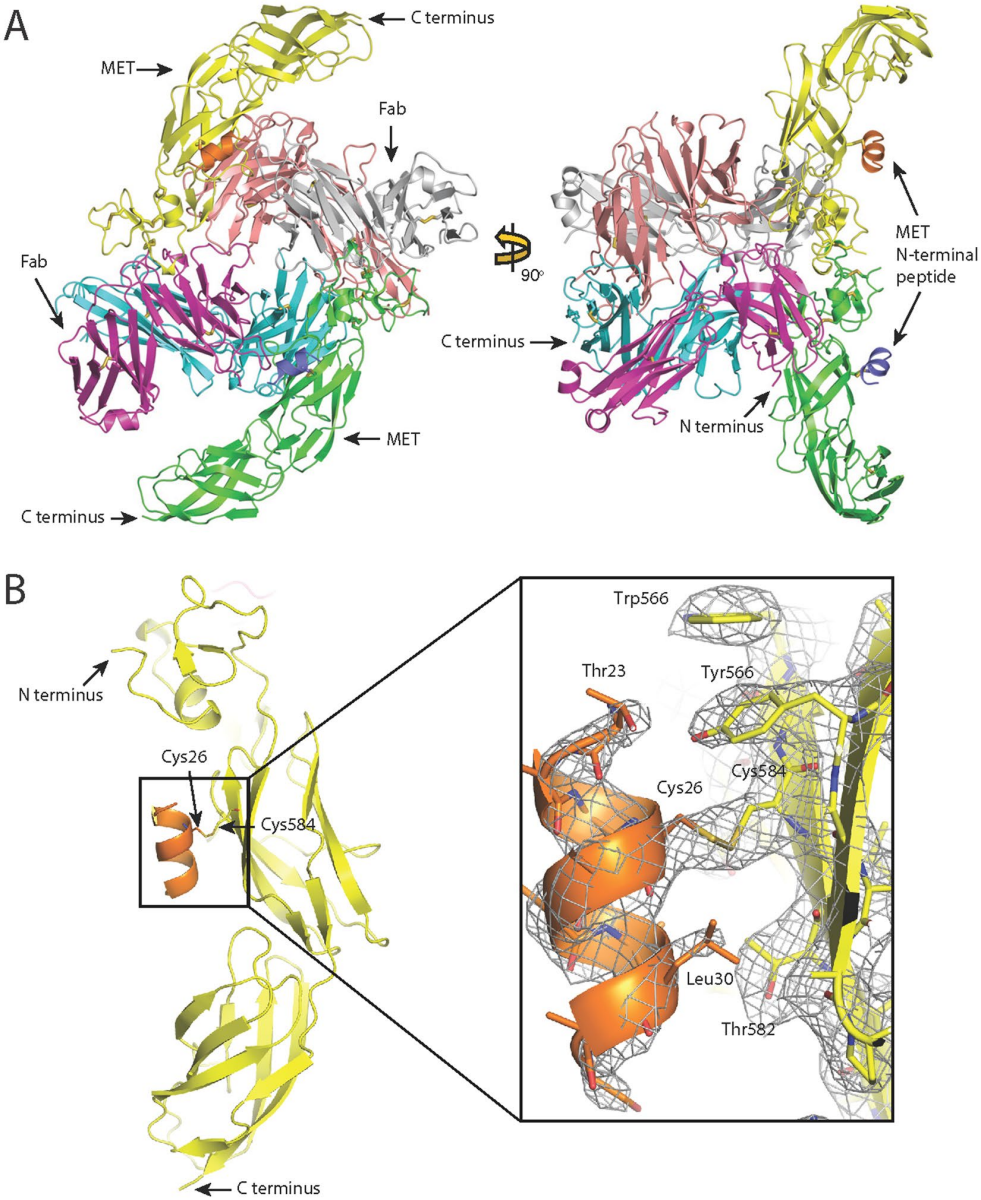
Crystal structure of the 107_A07-MET complex. **A**: Content of the asymmetric unit. The two views are related by a 90° rotation along the y axis. Complex molecule 1: MET receptor fragment (519–740) – green; MET receptor amino terminal peptide (22–32) – violet; 107_A07 Fab heavy chain – cyan; 107_A07 Fab light chain – magenta. Complex molecule 2: MET receptor fragment (519–740) – yellow; MET receptor amino terminal peptide (22–32) – orange; 107_A07 Fab heavy chain – salmon pink; 107_A07 Fab light chain – grey. The disulfide bridges are shown as sticks. The figure was generated with PYMOL^62^. **B**: The view showing the position of MET receptor amino-terminal peptide in orange (22–32) relative to MET receptor fragment in yellow (519–740). The disulfide bridge between residues Cys26 of the peptide and Cys584 of MET receptor fragment is shown in sticks. The zoomed in portion of the figure is showing the final 2Fo-Fc electron density map contoured at 1.1 sigma level.

**Figure 5:**
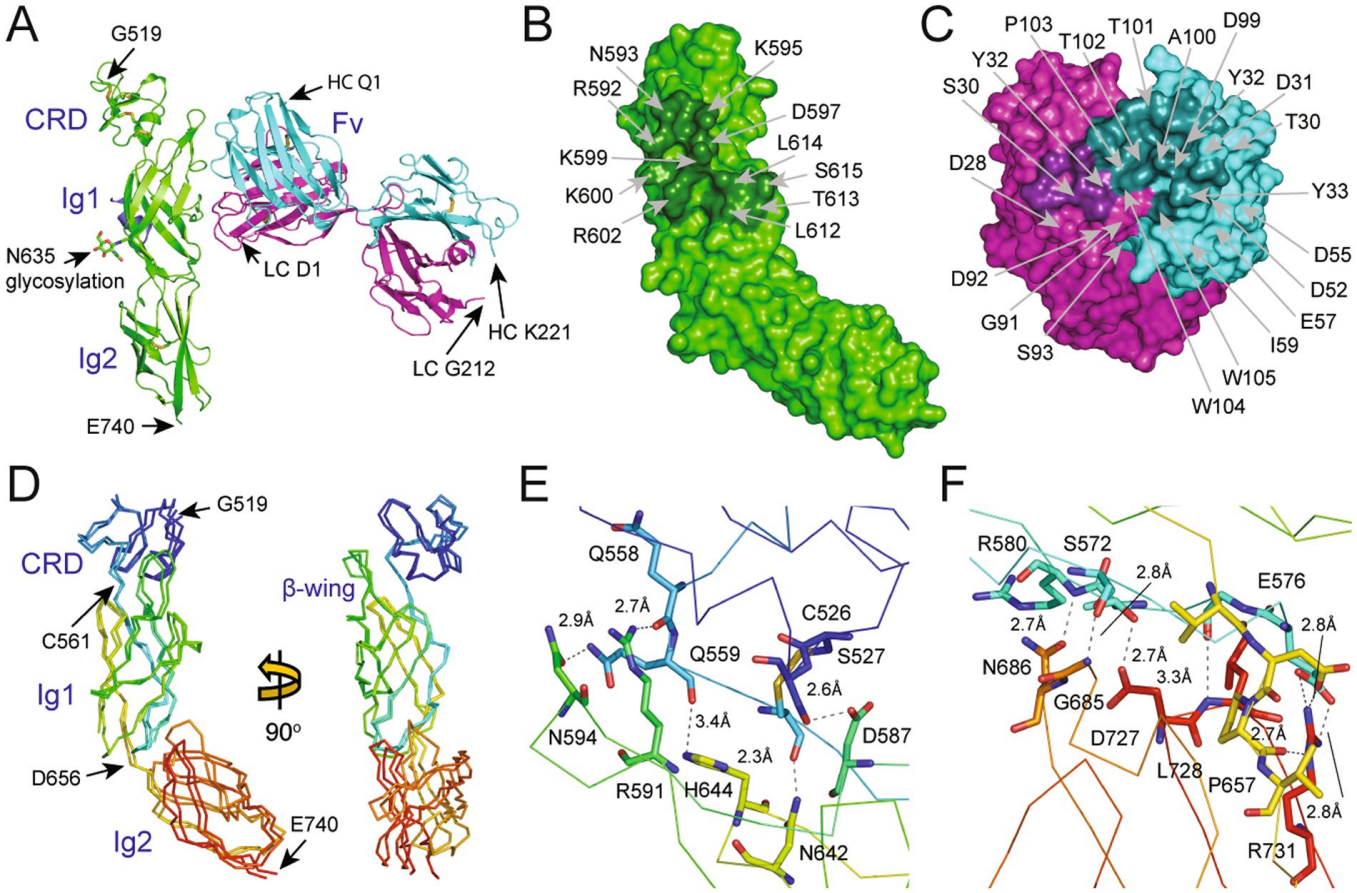
Structure of the complex between 107_A07 and MET519-740. (**A**) Ribbon diagram of complex 1 of the 107_A07 - MET519-740 structure showing the MET519-740 molecule (green) and the 107_A07 Fab on the right (heavy chain cyan, light chain purple). (**B**,**C**) Residues involved in contacts between MET519-740 (**B**) and 107_A07 Fab (VH domain in cyan, VL domain in purple). The picture highlights the polar and charged nature of the extensive VH-MET contacts (see Table S1 for further details). (**D**) Superposition of the structures of the MET fragment containing residues 519–740 from the structure of the 107_A07-MET complex (chain A) and the InlB-MET structure (PDB accession 2UZY, chain B). (**E** and **F**) Contacts between the CR domain and the IG1 domain (**E**) of MET519-740 and between the bottom of IG1 domain and the top of the IG2 domain (**F**). These two sets of contacts account for the rigid structure of the MET519-740 fragment of MET.

In order to define further the epitope and inhibitory mechanism of these antibodies, we determined the crystal structure of a complex formed by the 107_A07 Fab and a fragment of the MET ectodomain encompassing the small cysteine-rich domain and the upper stalk region (Ig domains 1 and 2). Initial crystallization experiments with complexes formed by the 107_A07 Fab and MET741 failed to yield diffraction grade crystals. We overcame this by exploiting a pepsin cleavage site (NGL|GCR) located in the linker connecting the SEMA and CR domains of MET, generating a receptor fragment containing the CR, Ig1 and Ig2 domains of MET, which contains MET residues 519–741. Crystals of the 107_A07 Fab – MET^519–741^ complex were obtained and diffracted to a maximum resolution of 2.6 Å at the European Synchrotron Radiation Facility (Beamline ID29, ESRF, Grenoble, France). The structure was solved by the Molecular Replacement method^43^ and refined to R_cryst_ and R_free_ values of 21.5% and 25.7% respectively (Table 1). The asymmetric unit contained two 107_A07 - MET^519–741^ complexes (Fig. 4A) that, when superposed on their MET-Ig1 domains, showed close alignment of the MET chain and VH and VL domains but approximately 60° rotation of the CH1 and CL domains. Complex 1, containing MET chain A and Fab chains H and L (Fig. 5A) is described below because the antibody-receptor interactions seen within this complex are more complete than the ones seen in complex 2. The model contains MET residues 519–740. We also observed unexpected electron density adjacent to Cys584 in the Ig1 domain (Fig. 4B). MALDI analysis of the crystallization complex and subsequent MS/MS analysis identified a peptide containing amino terminal residues of MET (residues 22–32, which include a single cysteine at position 26) attached to the complex by a disulphide bond. Together, these data indicate the presence of a disulphide bond between MET residues Cys26 and Cys584. Since MET^519–741^ was purified by enzymatic digestion of the MET741–107_A07 complex, it is uncertain whether the attachment occurred before or after digestion.

**Table 1:** Crystallographic data collection and refinement statistics.

	MET - 107_A07 Fab complex
**Data collection**	
Radiation Source, Beamline	ESRF, ID29
Wavelength (Å)	0.91376
Space group	P22_1_2_1_
Cell dimensions	
a, b, c (Å)	71.88 82.28 267.30
α, β, γ (°)	90, 90, 90
Resolution (Å)	48.95–2.60 (2.74–2.60)1
R_meas_^2^	11.1 (88.3)
<I/σ(I)>	11.7 (2.0)
Completeness (%)	99.0 (95.8)
Redundancy	5.5 (5.5)
No. of unique reflections	49,099 (6,685)
**Refinement**	
Resolution (Å)	48.94–2.60
No. of reflections:	
Total	48,940
Rfree set	2,000
R_cryst_^3^/R_free_^4^	21.5/25.7
Contents of asymmetric unit:	
Protein atoms	10,043
Solvent atoms	88
R.m.s deviations:	
Bond lengths (Å)	0.005
Bond angles (°)	0.999

^1^The statistics shown in parentheses are for the highest-resolution shell.

^2^ *R*_meas_ = (Σ _*hkl*_ [N/(N-1)]^1/2^ Σ _*i*_ |*I*_*i*_(hkl)−*I*_*mean*_(hkl)|)/Σ _*hkl*_ Σ _*i*_ *I*_*i*_(hkl), where N is redundancy.

^3^ *R*_cryst_ = Σ _*hkl*_ ||F_obs_(hkl)|−|F_calc_(hkl)||/Σ _*hkl*_ |F_obs_(hkl)|.

^4^ *R*_free_ is the same as *R*_cryst_ for a random subset not included in the refinement of about 4% of total reflection.

The VH and VL domains of 107_A07 bind the Ig1 domain of MET (Fig. 5B) and bury 555 Å^2^ and 243 Å^2^ respectively. The VH domain binds a discontinuous epitope including the tip of the β-hairpin (R592, N593 and K595), residues connecting the β-hairpin and the β-strand C (K599 and K600) and the tip of β-strand C (R602) (Fig. 5B and Supplementary Table S1) and involves an extensive network of polar contacts and salt bridges involving CDRs 1, 2 and 3 of the antibody heavy chain (Fig. 5C and Table S1).

The interactions of the VH domain with MET clarify the impact of the Y105W mutation that was found in each of the top 8 affinity matured clones. From the structure it is clear that the indole group of tryptophan creates an additional sidewall for the pocket formed by residues 99–105 of the heavy chain. This makes the pocket deeper and more complementary for the interactions with the antigen’s residue K599. The side chain of K599 is fully extended and inserts deep into the pocket, where the NH_2_ group creates an extensive hydrogen bonding network with the carbonyl groups of the backbone (Fig. S6). In contrast, the VL domain binds a linear epitope (L612, T613, L614, S615 and E616, a sequence corresponding to β-strand D of canonical E set domains) and makes limited contacts with MET predominantly via L3 (Fig. 5C and Table S1).

Within the 107_A07 Fab - MET complex, the conformation of the CR domain of MET relative to the Ig1-Ig2 fragment matches closely the conformation of the corresponding region of MET observed in the crystal structure of MET741 in complex with InlB321. InlB321 is a truncated fragment of InternalinB (InlB), a surface protein of *Listeria monocytogenes* that promotes binding and internalization of the bacterium through binding and activation of the MET receptor^44, 45^ (PDB entry 2UZY). InlB321 binds with high affinity to a primary site in the Ig1 domain of MET with its leucine-rich repeat central domain. It also binds with lower affinity to a secondary site in the SEMA domain with its interdomain repeat. Binding to both sites is essential for receptor activation^44, 45^.

In contrast to InlB321, 107_A07 appears to contact only the MET Ig1 domain; however, the C-alpha atoms for the CR-Ig1-Ig2 structure of the InlB-MET741 superpose closely with the corresponding MET domains in the 107_A07 structure (r.m.s.d. is ~1.4 Å from the MET molecule in complex 1 and ~2.0 Å for the MET molecule in complex 2 (Fig. 5D). Although extensive movements of the SEMA domain relative to the CR domain are enabled by the SEMA-CR domain linker^30, 37, 44, 45^, the structure of the MET CR-Ig1-Ig2 section reported here is rigid, due to extensive interactions between the top of Ig1 (notably the β-hairpin structure) and the CR domain (Fig. 5E), in addition to a second set of interactions between Ig1 and Ig2 (Fig. 5F).

## Discussion

We have isolated and affinity matured a novel phage-derived anti-MET antibody, 7A2/107_A07, which competes for binding with both MET ligand HGF/SF and the HGF/SF fragment NK1. Antibody 7A2/107_A07 inhibited HGF/SF-induced cell migration and DNA synthesis *in vitro*, endothelial cell tubulogenesis in a co-culture assay and tumor growth *in vivo* in a xenograft model. Biochemical analysis and structural determination demonstrated that the antibody binds to the Ig1 domain, in contrast to HGF/SF, which binds to the SEMA domain.

**Figure 6:**
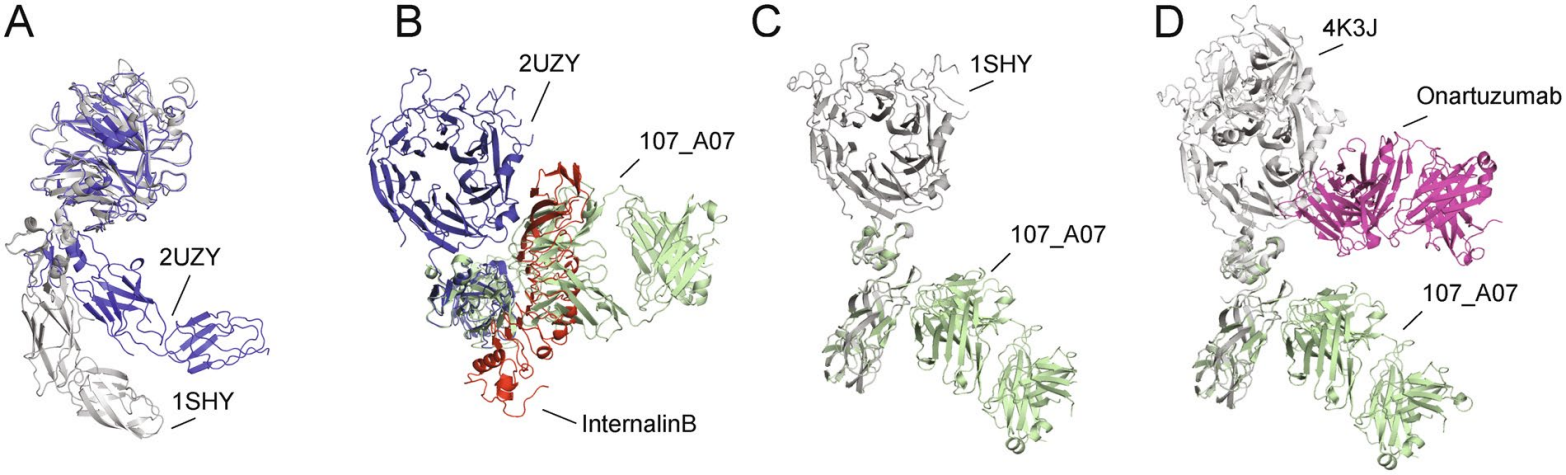
Comparison of the 107_A07 Fab with compact (InlB-bound) and extended (HGF/SFβ-bound) structures of the MET ectodomain. (**A**) The compact (blue) and extended (grey) conformations of extracellular MET (amino acids 25–741). The structure of compact MET is from PDB accession 2UZY (chain B), (complex of MET with InlB); the structure of MET in extended conformation is from PDB accession code 1SHY (HGF/SF beta chain in complex with SEMA domain of MET). (**B**) Superposition of the MET-107_A07 Fab complex (shown in green) with MET in compact conformation (2UZY), obtained by superposing the CR domains. The MET CRD-Ig1-Ig2 domains are oriented perpendicular to the image. The InternalinB structure is shown in red. (**C**) Superposition with MET in extended conformation (1SHY). The position of Ig1-Ig2 was obtained as described in (**B**). (**D**) Superposition with MET in extended conformation (4K3J, complex of MET and the Fab fragment of onartuzumab). The onartuzumab structure is shown in magenta.

The domain architectures of MET and its homologue RON differ from those of other receptor tyrosine kinases (RTK)^46^ and are evolutionary and structurally related to the plexins and their semaphorin ligands^47^. Cryo-EM and small angle X-ray scattering (SAXS) analysis of the soluble MET ectodomain^42^ and several crystal structures^30, 37, 44, 45^ have clearly established that the first four extracellular domains of MET (the SEMA, CR and the first two Ig domains) can adopt a more compact or a more extended conformation as a result of rotation and translation of the CR domain relative to the SEMA domain (Fig. 6A). The present study revealed, unexpectedly, that the CR-Ig1-Ig2 domains of MET form a rigid body and this indicates that the first 741 residues of MET contain a single hinge, located between the SEMA and CR domains.

The rigid structure of the CR-Ig1-Ig2 fragment has profound implications for the mechanism of MET inhibition by 107_A07. Crystal structures of soluble fragments of MET in complex with the SPH domain of HGF/SF^30, 37^ or InlB^44, 45^ have shown that the CR domain can adopt two main orientations relative to the SEMA resulting in a compact (PDB entry 2UZY) or extended (PDB entries 1SHY and 4K3J) arrangement of the SEMA, CR, Ig1 and Ig2 domains (Fig. 6A). The compact architecture prevails in solution as shown by SAXS experiments^42^ although the molecular mechanism for this apparent restriction in hinge flexibility is not known.

One other crystal structure of a MET-antibody complex is available (the Fab fragment of onartuzumab in complex with MET567 and the SPH domain of HGF/SF (4K3J)) and shows that onartuzumab binds an area of the SEMA domain that overlaps with the secondary binding site of InlB^37^. Onartuzumab competes for binding with both HGF/SF and NK1^37^ and it has been concluded that the binding sites of HGF/SF, InlB and onartuzumab on the SEMA domain overlap in full or in part^37^. In contrast, 107_A07 binds an epitope on the Ig1 domain of MET that overlaps with the primary binding site of InlB, yet 107_A07 also competes with HGF/SF and NK1 for MET binding.

Structural alignment of the MET-InlB complex and the MET-107_A07 complex indicates that when MET is in the compact conformation, the binding sites for InlB on the MET SEMA and 107_A07 on the Ig1 domain are spatially proximate, despite their location on separate domains (Fig. 6B and Fig. S7). While an allosteric mechanism cannot be ruled out, steric competition between HGF/SF and 107_A07 when MET is in the compact conformation would readily explain the observed biochemical competition between HGF/SF and 107_A07. The compact conformation of MET predominates in solution^42^ and occurs in the crystal structure of the InlB-MET741 complex^44, 45^. Our data further support a role for this conformation in HGF/SF-induced MET signaling.

Our data also demonstrate that the Ig1 residue Cys584 is capable of forming a disulphide bond external to the Ig1 domain. A putative disulphide linkage between Cys584 (Ig1 domain) and Cys26 (N-terminal) has been postulated and discussed previously^42, 44, 48^ but to our knowledge this is the first time that experimental evidence for this bond has been observed. Attachment of Cys584 to the N-terminal peptide of MET may have occurred before or after proteolysis; whether this disulphide bond exists in intact, full-length MET remains to be determined. The possibility is intriguing as such a bond, if it exists, may go some way towards explaining why the compact arrangement of the SEMA, CR, Ig1 and Ig2 domains has been reported to dominate over the extended conformation in SAXS experiments^42^.

The pivotal roles of HGF/SF and MET in cancer progression and metastasis have led to considerable expectation that agents blocking HGF/SF-MET signaling could have a strong impact in the therapy of metastatic tumors and led to a major effort in the design, synthesis and development of small molecule inhibitors of the MET kinase^49^ as well as blocking antibodies directed against the ligand HGF/SF^50, 51^ and the MET extracellular region^37, 52–56^. Several anti-MET antibodies progressed to clinical trials, including ABT-700^57^, LY2875358^58^, ARGX-111 and onartuzumab. The development of a number of anti-MET antibodies with antagonistic activity has highlighted diverse mechanisms for receptor inhibition. For instance, onartuzumab^37^ and anti-MET CE-355621^54^ inhibit HGF/SF binding, MET activation and xenograft growth. Anti-MET LMH 87^53^ does not inhibit ligand binding but promotes receptor degradation and anti-MET F46 inhibits ligand binding and promotes receptor degradation^59^. An anti-MET nanobody (a single VH domain) inhibits MET activation and DNA synthesis in myeloma cells^60^ and a recent collection of llama anti-MET antibodies highlights multiple mechanisms of receptor inhibition^56^. These multiple pathways of receptor inhibition reflect the complex structural basis of MET signaling^30, 44, 45^. An anti-MET anticalin (PRS-110) with MET antagonistic activity has also been developed^61^. Interestingly, PRS-110 binds both to a loop in the SEMA domain in close proximity to the K1 binding site as well as the β-wing of the Ig1 of MET suggesting that the PRS-110 and 107_A07 epitopes may be closely related. While crystallographic analysis will be required in order to define more accurately the epitopes of a number of other anti-MET antibodies under development for therapy, the structures of onartuzumab^37^ and 107_A07 (this work) in complex with MET offer initial insights into mechanisms of MET inhibition. The unusual mechanism of MET inhibition by 107_A07, involving biochemical competition for ligand binding despite clear separation of binding footprints, contrasts with that of onartuzumab and illuminates the variety of potential mechanisms through which antibody-mediated MET inhibition can be achieved.

## Electronic supplementary material

Supplementary Information
