# Supplementary material for: Characterization and structural determination of a new anti-MET function-blocking antibody with binding epitope distinct from the ligand binding domain
Source: Sci Rep. 2017 Aug 21;7:9000. doi: 10.1038/s41598-017-09460-2 (PMC5567289; doi:10.1038/s41598-017-09460-2)
Supplement: Supplementary file 1 — Supplementary Information [file 41598_2017_9460_MOESM1_ESM.pdf]

Supplementary Information (SI)

Characterization and structural determination of a new anti-MET function-blocking antibody with binding epitope distinct from the ligand binding domain.

Danielle M. DiCara[1,2,♯], Dimitri Y. Chirgadze[3], Anthony R. Pope[4], Aneesh Karatt-Vellatt[4], Anja Winter[3,‖], Peter Slavny[4], Joop van den Heuvel[5], Kothai Parthiban[4], Jane Holland[6], Len C. Packman[3], Georgia Mavria[7], Jens Hoffmann[8], Walter Birchmeier[6], Ermanno Gherardi[1,2,9,*] and John McCafferty[3,4,*]

1 MRC Centre, Hills Road, Cambridge CB2 2QH, UK

2 Department of Oncology, University of Cambridge, Cambridge Biomedical Campus, Cambridge CB2 0XZ, UK

3 Department of Biochemistry, University of Cambridge, 80 Tennis Court Road, Cambridge CB2 1GA, UK

4 IONTAS Ltd, Babraham Institute, Babraham, Cambridgeshire CB22 3AT

5 Helmholtz Zentrum für Infektionsforschung, Inhoffenstraße 7, 38124 Braunschweig, Germany

6 Max Delbrueck Center for Molecular Medicine (MDC) in the Helmholtz Association, 13125 Berlin, Germany

7 Leeds Institute of Cancer and Pathology, University of Leeds, St James' University Hospital, Beckett Street, Leeds LS9 7TF, UK

8 Experimental Pharmacology & Oncology Berlin-Buch GmbH, Robert-Rössle-Str. 10, 13125 Berlin-Buch, Germany

9 Division of Immunology and General Pathology, Department of Molecular Medicine, 1 via A Ferrata, 27100 Pavia, Italy

♯ Current address: Genentech Inc., South San Francisco, 94080, USA

‖ Current address: Faculty of Natural Sciences, Keele University, Staffordshire ST5 5BG, UK

* Joint senior authors. Correspondence to: egherard@unipv.it (EG) or jmc@iontas.co.uk (JMC)

Supplementary Materials & Methods

Protein production and purification: Soluble MET741 protein [1] was produced from CHO Lec3.2.8.1 grown in a bioreactor at 32° C in serum-free ProCHO5 medium. The culture supernatant was concentrated and dialysed against 25 mM Na-phosphate pH 7.4, 150 mM NaCl using crossflow and a cartridge with 30 kDa cutoff (Millipore). The protein was purified by affinity chromatography over NiNTA superflow (Qiagen) followed by MonoS (GE Healthcare) using a NaCl gradient in 50 mM MES, pH 6.0. 107_A07 and D1.3 Fabs were produced by PEI-mediated transient transfection of suspension HEK293F cells (Invitrogen) grown in serum-free FreeStyle293 medium with Valproic Acid (Sigma) added to 4mM following transfection. Fab proteins were purified using affinity resins KappaSelect and/or GammaBind Plus (GE Life Sciences). For Cell Cycle Analysis, 107_A07 and D1.3 Fabs were further purified by gel filtration chromatography (Superdex 200 10/300 (GE Life Sciences). Unless stated otherwise, 7A2 scFv was produced in *Pichia pastoris* and purified by Ni-NTA chromatography followed by gel filtration.

FAb PEGylation: Recombinant Fab was reduced using 5mM TCEP-HCl (Tris (2-carboxymethyl) phosphine hydrochloride prior to pegylation. The TCEP was then removed using a Zeba (Pierce Thermo) desalting column[2] and then dialysed into 100mM phosphate / 2mM EDTA pH 6. The antibody was pegylated with maleimide activated PEG (MW 20,000) with either a (CH2)2 linker between the PEG and the maleimide group (Sunbright ME-200 MAOB or with a (CH2)5 linker (Sunbright ME-200MA3), both supplied by NOF Europe. PEG, PEG-Fab and free PEG were monitored by PAGE, staining for protein with Coomassie blue and for PEG with barium chloride and Iodine [3]. The majority of unconjugated PEG was removed by gel filtration (Superdex 200 16/60, GE Life Sciences) and ultrafiltration (Amicon Ultra-15, 30kD NMWCO, Millipore).

Isolation and affinity maturation of functional MET-blocking antibodies by phage display: Two rounds of biopanning were performed on immunotubes coated with recombinant MET extracellular domain (MET928) with a scFv phage library described before [4]. Light chain shuffling was then performed on the output by cloning the resulting VH gene pool back into the original scFv phage library [5]. The chain-shuffled library (10^{9} clones) was exposed to

biotinylated MET928 in solution and phage-MET complexes isolated with streptavidin-coated dynabeads. MET-bound phage were eluted either by trypsin treatment (expected to elute all specifically bound phage antibodies by cleaving a trypsin-sensitive site located between the scFv and the fused gIII), or alternatively by exposure to 2μM HGF/SF. Following two rounds of selection with trypsin elution or three rounds of selection with HGF/SF elution at rounds 2 and 3, the scFv pools were cloned into expression vector [6]. Clone 7A2 was obtained from a selection track involving biotinylated MET928 concentrations of 100 nM in round 1, 10 nM in round 2 and 1 nM in round 3, with HGF/SF elution in rounds 2 and 3. Small-scale expressions were performed in BL21 (DE3) bacteria in 96-well format. Approximately 960 supernatants were screened directly for inhibition of HGF/SF-induced scatter of BxPC-3 human pancreatic cancer cells. Selected antibody clones were expanded, purified and screened for inhibition of HGF/SF-induced SKOV3 human ovarian cancer cell migration in the modified Boyden chamber assay. 7A2 was selected for further study following complete blockade of HGF/SF-induced SKOV3 migration during screening.

Affinity maturation of 7A2: Display libraries were constructed where the CDR3 regions of the V gene heavy chain (VH) and light chain (VL) of 7A2 were diversified using oligonucleotide-directed mutagenesis. Mutagenic primers based on the 7A2 sequence were designed with contiguous blocks of 5-6 randomised amino acids (using NNS codons) using 3 overlapping primers to randomise the 11 amino acids of the VH CDR3 and 2 primers for the 9 amino acids of VL CDR3. Libraries of 6.7 x 10^8 heavy chain variants and 5.5 x 10^8 light chain variants were constructed. Sequencing confirmed that randomization was achieved in the appropriate positions. One round of selection was carried out on the separate VH and VL libraries to remove non-functional clones in each library. The selected populations were then re-combined to create a final library of 1.7 x 10^9 clones. Stringent selections were carried out on this composite library using a biotinylated form of the MET-928 extracellular domain down to 10pM concentration. All improvements came from VH CDR3 mutagenesis where the mutagenic primers were:

VH 3.1 5’: ACGGCCGTGTATTACTGTGCAACANNSNNSNNSNNSNNSTACTACGGTATGGACGTCTGGGGC
VH 3.2 5’: ACGGCCGTGTATTACTGTGCAACAGATGCTACGNNSNNSNNSNNSNNSATGGACGTCTGGGGCCAAGGAACC
VH3.3 5’: GCAACAGATGCTACGACGCCCTACNNSNNSNNSNNSNNSTGGGGCCAAGGAACCCTGGTCAC

HGF/SF-induced cell migration and cell cycle analysis: FAbs were analysed for inhibition of HGF/SF-induced cell migration using a modified Boyden chamber assay (AC96 Migration Chamber; Neuroprobe). Lower chambers containing 30pM HGF/SF and antibodies diluted in Assay Media (a 1:1 mixture of PBS and RPMI, 0.25% BSA) were separated from upper chambers by a porous membrane (8µm, PVP-free) that had been coated with 100 µg/ml Collagen (Purecol, Nutacon) for 2-3 hours at room temperature. SKOV-3 or U87MG cells were labelled with the fluorescent dye Calcein AM (Life Technologies) and 25,000 cells then added to each upper well. After four hours at 37°C the apparatus was disassembled and non-migrated cells removed from the membrane by gentle wiping with cotton wool. Cell migration was assessed by quantification of the residual fluorescence, indicative of migrated cells, on a Typhoon instrument (GE Life Sciences), using excitation/emission settings of 488nm/526nm respectively. Data were analysed with ImageQuant software and background fluorescence subtracted. For cell cycle analysis, U87MG human glioblastoma cells were plated in full media at 100,000 cells/well in a 12-well tissue culture dish and allowed to adhere overnight. Cells were serum-starved for 48 hours prior to a 24 hour incubation with 300pM HGF/SF with or without 0.9µM 107_A07 FAb or 1µM D1.3 FAb. Cells were trypsinised, fixed, stained with propidium iodide in the presence of RNAse and analysed by flow cytometry according to standard procedures.

Endothelial cell tubulogenesis assay: *In vitro* angiogenesis assay was performed using the modified co-culture assay as described previously [7]. Reagents and cell lines were purchased from TCS Cellworks. Briefly 8,000 fibroblast cells were seeded in each well of a gelatin-coated 8-well chamber slides. After 5-7 days, 5,000 HUVECs were seeded on to the confluent fibroblasts and D1.3 and 107_A07 antibodies (200nM) were added to the cells. On day 3 and 5 medium was changed and replaced with fresh antibodies. The co-cultures were fixed on day 10 and stained for CD31. Staining was done using CD31 tubule staining kit (ZHA-1225) and the bright-field images were captured with a 4x objective (Nikon Diaphot200). Number of tubules was counted manually from 10 fields for each well and the field area was measured using AngioSys 1.0 imaging software.

Co-crystallisation of MET/Fab complex: His-tagged MET741 and untagged 107_A07 FAb were co-incubated for 140 minutes at a 1:1.6 molar ratio prior to addition of EndoHf

deglycosidase and Pepsin and further incubation for 48 hours at 4˚C. Cleaved, digested MET-Fab complex was purified on Ni-NTA agarose (Qiagen), eluted with 200 mM EDTA and exchanged into crystallisation buffer (25mM Tris pH7.4, 200mM sodium chloride, 7.5% v/v glycerol) by gel filtration (Superdex 200 16/60 column, GE Life Sciences). Prior to gel filtration the mixture was exposed briefly to Dextrin-Sepharose (GE Life Sciences) as a precaution against contaminating EndoHf, which elutes at a similar volume to the digested MET519-740-Fab complex. Purified complex eluted essentially as a single peak and was concentrated to approximately 5.9 mg/ml with a 10kD MWCO Amicon Ultra-15 centrifugal filter device (Millipore). Sitting-drop vapour diffusion crystallisation trials (total volume 200nl, 1:1 ratio of protein:precipitant) were set up in MRC2 crystallisation plates using the Phoenix crystallisation robot (Art Robbins Instruments, Inc). Crystallisation plates were incubated at 19°C and monitored with a ROCK Imager 500 (Formulatrix, Inc) automated imaging system. Following initial crystallisation hits with the Morpheus protein crystallisation screen (Molecular Dimensions), 1µl+1µl drops were manually set up with Morpheus condition A9 (10% PEG 20,000, 20% PEG 550-MME, 0.1M Trizma/Bicine pH 8.5, 0.03M Magnesium Chloride, 0.03M Calcium Chloride) in a 24-well Intelliplate (Art Robbins Instruments, Sunnyvale, CA, USA). Prior to contact with the protein, the precipitant was supplemented with 5% (v/v) glycerol. Glycerol was not added to the reservoir. Crystals appeared within 24 hours and were harvested on day seven, incubating briefly for approximately 5-10 seconds in Morpheus condition H8 (12.5% w/v PEG 1000, 12.5% w/v PEG 3350, 12.5% v/v MPD, 0.2 M sodium l-glutamate, 0.2 M dl-alanine, 0.2 M glycine, 0.2 M dl-lysine HCl, 0.2 M dl-serine, 0.1 M MOPS/HEPES-Na pH 7.5) supplemented with 20% glycerol prior to freezing in liquid nitrogen.

X-ray diffraction data collection & structure solution: X-ray data collection experiments were performed at 100K temperatures at the European Synchrotron Radiation Facility (ESRF, Grenoble France), beamline ID29. The crystals diffracted to a maximum resolution of 2.6 Å (the resolution cut-off level was set to the resolution shell were the average I/sigma of the reflections is still greater than 2). A total of 150 degrees of data were collected at 0.05 degree oscillation angle. The crystals belonged to the $P22_12_1$ space group and contained two molecules of the MET/Fab complex (approximate molecular weight of the complex 71.3 kDa) in the asymmetric unit. This results in approximately 56% crystal solvent content (Matthews

Coefficient of 2.77). All diffraction data were indexed, scaled and merged using XDS Suite [8]. Crystallographic data collection statistics are shown in Table 1. The crystal structure of the MET/FAb complex was solved using the Molecular Replacement (MR) method. The positions of individual domains, i.e. the constant and variable domains of the heavy and the light chains of Fab and the two immunoglobulin-like (Ig) domains of the MET receptor fragment within the asymmetric part of the unit cell were identified. All MR calculations were performed in PHASER (part of PHENIX software suite distribution, version 1.7.2-869). The MR search probes included the constant and variable domains of the H and the L chains of Fab fragment crystal structure (PDB-ID: 1RZ7) and the two Ig domains from the crystal structure of MET in complex with the *L monocytogenes* invasion protein InlB (PDB-ID: 2UZY). All positional solutions of the domains (even for the first domain) had the translation function Z-score values above 8, indicating the correctness of the MR solution. The refinement calculations were performed in PHENIX while manual rebuilding was performed in COOT. The first round of refinement calculations with the obtained model caused a significant drop in R/R_{free} values by about 6% each, reaching the final values of 33.4% and 37.4%, respectively thus indicating the correctness of the obtained MR solution. After 5 rounds of manual rebuilding and refinement the in R/R_{free} values are 21.5% and 25.7%, respectively.

Supplementary Tables & Figure Legends

MET

SEQ	AA	HS	ASA	BSA
PHE	590		35.0	0.6
ARG	592	HS	135.0	106.0
ASN	593	H	136.4	57.7
LYS	595	H	128.3	42.1
ASP	597		37.3	19.4
LEU	598		15.5	1.3
LYS	599	HS	145.9	139.4
LYS	600	S	86.6	69.4
ARG	602	S	122.9	74.2
LEU	614		99.6	48.1

107_A07 H

SEQ	AA	HS	ASA	BSA
THR	30	H	47.8	3.8
ASP	31	H	91.0	47.2
TYR	32		64.2	16.1
TYR	33		72.2	67.5
LEU	50		6.5	0.7
ASP	52	S	22.7	21.9
GLU	54		118.0	5.3
ASP	55	S	98.0	15.1
GLU	57	S	92.7	43.1
ILE	59		83.5	23.8
ASP	99	S	10.1	7.1
ALA	100	H	19.3	2.1
THR	101	H	97.9	27.8
THR	102		55.7	9.3
PRO	103		145.4	50.8
TYR	104		147.1	16.5
TRP	105		145.0	59.7

Hydrogen Bonds

107_A07 H	MET	Å
ASP31 (O)	ARG592 (NE)	2.7
THR30 (O)	ARG592 (NH1)	3.0
ASP31 (O)	ASN593 (ND2)	3.1
THR101 (O)	LYS595 (NZ)	2.3
ALA100 (O)	LYS599 (NZ)	2.7

Salt Bridges

107_A07 H	MET	Å
ASP52 (OD1)	ARG592 (NH1)	2.4
ASP52 (OD2)	ARG592 (NH1)	3.7
ASP52 (OD1)	ARG592 (NH2)	3.3
ASP52 (OD2)	ARG592 (NH2)	3.0
ASP99 (OD1)	LYS599 (NZ)	3.5
ASP99 (OD2)	LYS599 (NZ)	2.6
ASP52 (OD2)	LYS600 (NZ)	2.8
ASP55 (OD1)	LYS600 (NZ)	3.3
ASP55 (OD2)	LYS600 (NZ)	2.7
GLU57(OE1)	LYS600 (NZ)	2.8
GLU57(OE1)	ARG602 (NE)	3.8
GLU57(OE2)	ARG602 (NE)	2.8
GLU57(OE1)	ARG602 (NH2)	3.2
GLU57(OE2)	ARG602 (NH2)	3.7

ARG	580		81.5	2.2
LYS	599		145.9	0.3
ARG	602		122.9	14.1
THR	611		86.5	31.2
LEU	612		45.0	21.7
THR	613		35.1	23.6
LEU	614	H	99.6	51.5
SER	615	H	107.7	77.3
GLU	616		77.5	12.4

107_A07 L

SEQ	AA	HS	ASA	BSA
GLN	27		91.0	6.4
ASP	28	H	101.0	20.3
ILE	29		1.0	1.0
SER	30		52.7	15.8
TYR	32		124.7	36.6
GLY	91		40.7	11.1
ASP	92	H	60.3	57.9
SER	93		47.8	32.2
PHE	94		179.7	40.4

Hydrogen Bonds

107_A07 L	MET	Å
ASP28 (O)	SER615 (OG)	3.7
ASP 92 (O)	LEU614 (N)	3.2
ASP 92 (OD1)	SER615 (N)	2.7
ASP 92 (OD1)	LEU614 (N)	3.0
ASP 92 (OD2)	SER 615(OG)	2.8

Abbreviations: H: hydrogen bond, S: salt bridge, ASA: available surface area, BSA: buried surface area.

Supplementary Table S1

Contacts between the MET receptor fragment 519-741 and the heavy (H) and light (L) chains of the 107_A07 Fab (complex 1).

A

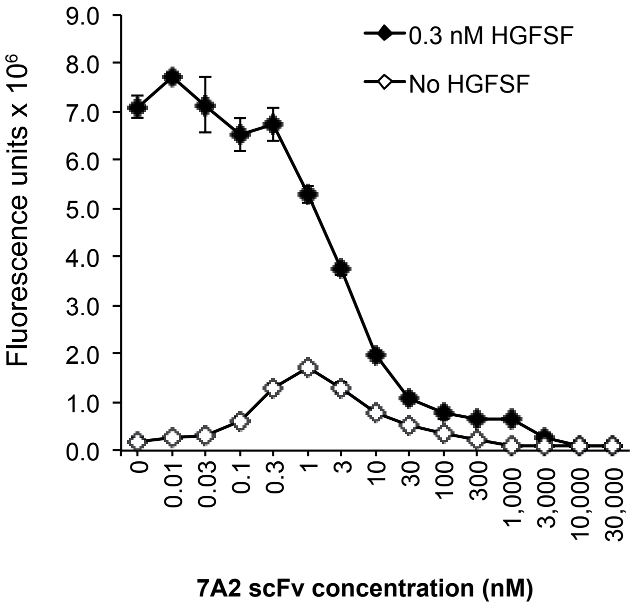

B

7A2

```
CDR L1:   QASQDISNYLN
CDR L2:   DASNLET
CDR L3:   QQGDSFPLT

CDR H1:   DYYMH
CDR H2:   LVDPEDGETIYAEKFQ
CDR H3:   DATTPYYGMDV
```

107_A07

```
CDR H3:   DATTPYWGMMW
```

Fig. S1. The scFv 7A2 inhibits HGF/SF-induced cell migration.

(**A**) Effect of 7A2 scFv on migration of SKOV3 cells in the presence or absence of 300pM HGF/SF in a modified Boyden chamber assay. Data represent mean ± standard deviation of triplicate wells. (**B**) Amino acid sequences of 7A2 and 107_A07 CDRs. 107_A07 CDR sequences did not differ from 7A2 except for the heavy chain CDR3; residues that differ in 7A2 and 107_A07 are underlined. ScFv for this experiment was produced in E. coli[4].

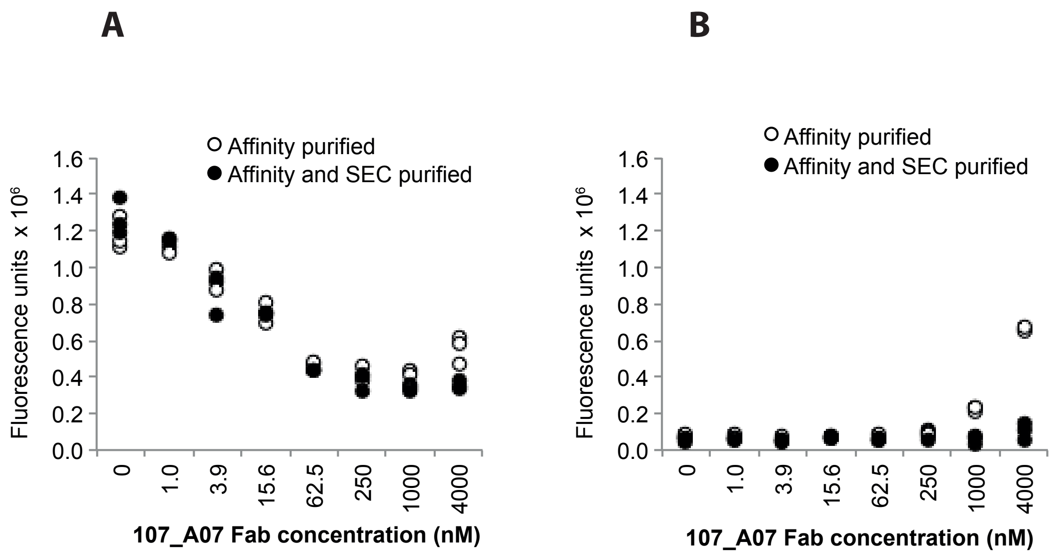

Fig. S2. Purification by size exclusion chromatography reduces agonistic behaviour of high concentration 107_A07 Fab.

Effect of 107_A07 Fab on migration of SKOV3 cells in the presence (A) or absence (B) of 30pM HGF/SF. Empty circles represent affinity-purified Fab; filled circles represent affinity-purified Fab purified additionally by size exclusion chromatography. Replicates are shown individually (n=2-3; wells lacking complete contact due to air at the membrane were excluded).

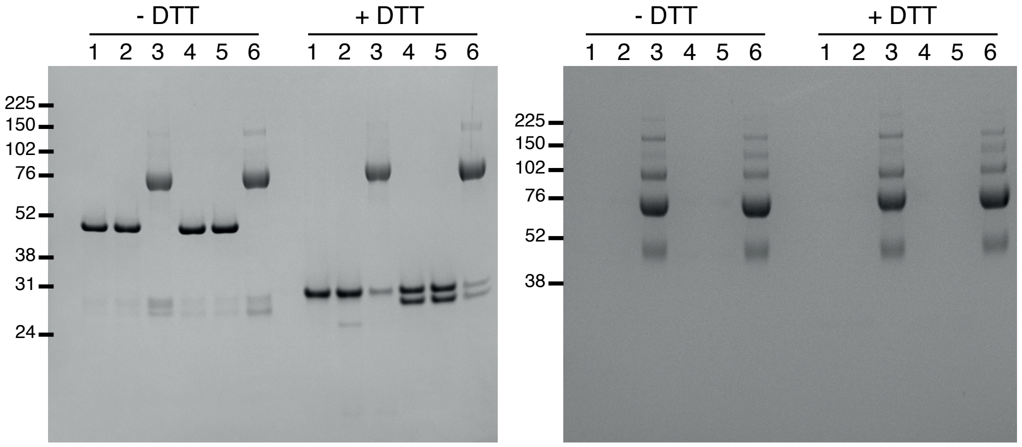

Fig. S3. SDS-PAGE analysis of native and pegylated Fabs.

Native and PEGylated Fabs were analysed by SDS-PAGE in the presence or absence of 100 mM DTT. Gels were stained either (A) with InstantBlue (Expedeon) to detect protein or (B) according to the method of Kurfurst *et al.* [3] to detect PEG. Lane 1: 107_A07 batch 1, lane 2: 107_A07 batch 2, lane 3: PEG-107_A07, lane 4: D1.3 batch 1, lane 5: D1.3 batch 2, lane 6: PEG-D1.3. PEGylated Fabs lost sensitivity to DTT and migrated more slowly than non-PEGylated Fab chains.

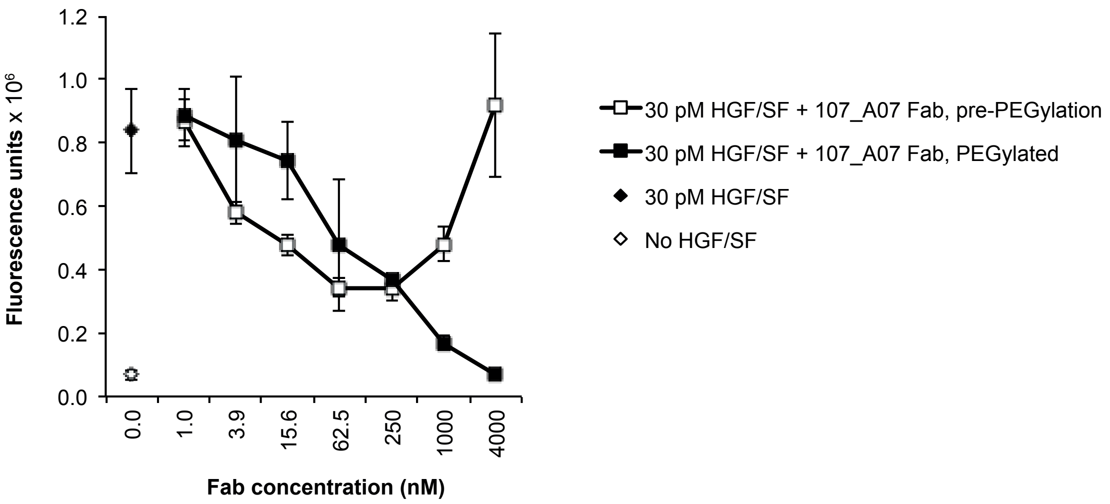

Fig. S4. Recovery of inhibitory activity by PEGylation of 107_A07 Fab.

The impact of 107_A07 Fab before and after PEGylation on migration of SKOV3 cells in the presence of 30 pM HGF/SF was compared. Data represent mean ± standard deviation of triplicate wells.

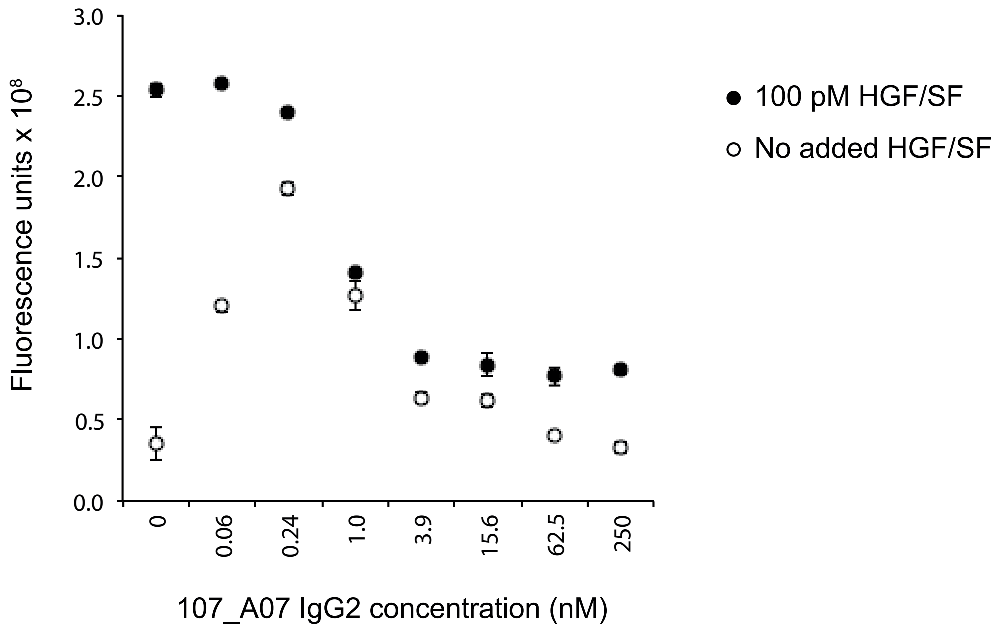

Fig. S5. Inhibitory and agonistic effects of 107_A07 IgG on SKOV3 migration. Antibody titration: data represent mean ± range of duplicate wells. Control wells (0 nM 107_A07 IgG): mean ± standard deviation of six wells.

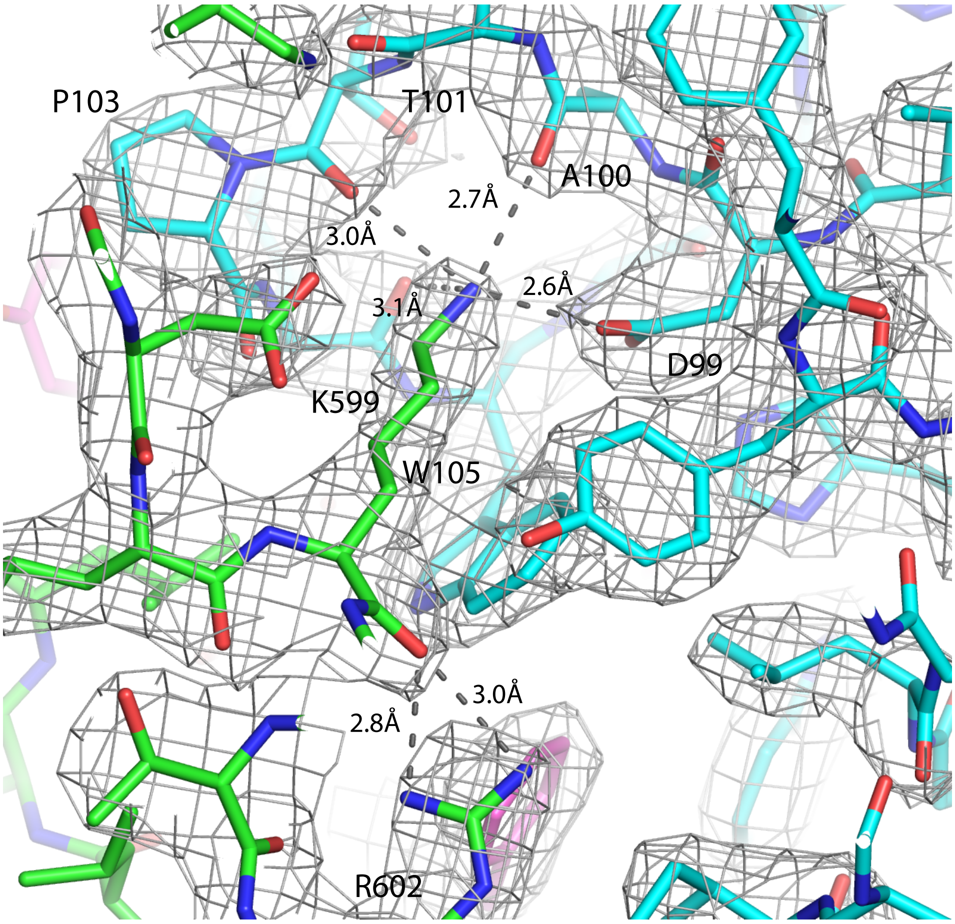

Fig. S6. Multiple hydrogen bonds formed by MET Lysine 599.

The affinity matured clones showing greatest improvement all had a Y105W mutation in VH CDR3. This supports an extended structure for K599 of MET allowing multiple hydrogen bonds to form.

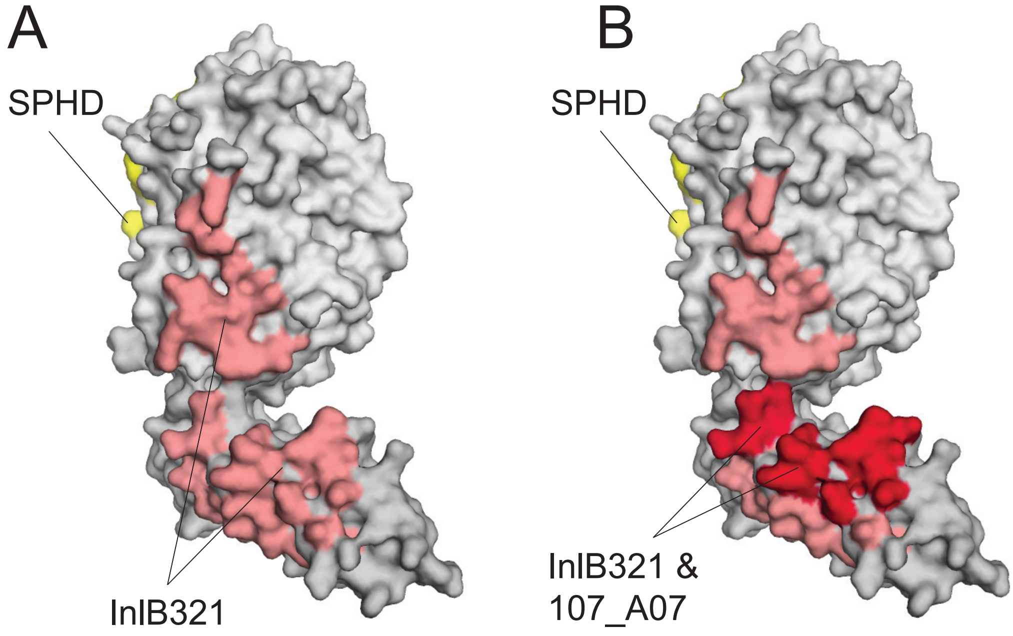

Fig S7. The 107_A07 epitope is contained in the InlB321 binding site.
(**A** and **B**) Comparison of the binding sites of InlB321 (salmon) (A) and the 107_A07 epitope (red). The latter is included in the binding site of InlB321. The figure was generated with PYMOL [9].

SUPPLEMENTARY REFERENCES:

1 Gherardi, E. *et al.* Functional map and domain structure of MET, the product of the c-met protooncogene and receptor for hepatocyte growth factor/scatter factor. *Proc Natl Acad Sci U S A* **100**, 12039-12044, (2003).

2 Shafer, D. E., Inman, J. K. & Lees, A. Reaction of Tris(2-carboxyethyl)phosphine (TCEP) with maleimide and alpha-haloacyl groups: anomalous elution of TCEP by gel filtration. *Anal Biochem* **282**, 161-164, (2000).

3 Kurfurst, M. M. Detection and molecular weight determination of polyethylene glycol-modified hirudin by staining after sodium dodecyl sulfate-polyacrylamide gel electrophoresis. *Anal Biochem* **200**, 244-248, (1992).

4 Schofield, D. J. *et al.* Application of phage display to high throughput antibody generation and characterization. *Genome Biol* **8**, R254, (2007).

5 Dyson, M. R. *et al.* Mapping protein interactions by combining antibody affinity maturation and mass spectrometry. *Anal Biochem* **417**, 25-35, (2011).

6 Martin, C. D. *et al.* A simple vector system to improve performance and utilisation of recombinant antibodies. *BMC Biotechnol* **6**, 46, (2006).

7 Hetheridge, C., Mavria, G. & Mellor, H. Uses of the in vitro endothelial-fibroblast organotypic co-culture assay in angiogenesis research. *Biochem Soc Trans* **39**, 1597-1600, (2011).

8 Kabsch, W. Xds. *Acta Crystallogr D Biol Crystallogr* **66**, 125-132, (2010).

9 DeLano, W. L. *The PyMOL Molecular Graphics System*. (DeLano Scientific, 2002).
